# The gender-related variability in the pharmacokinetics and antiplasmodial activity of naphthoquine in rodents

**DOI:** 10.1186/s12936-020-3153-8

**Published:** 2020-02-13

**Authors:** Yuewu Xie, Huixiang Liu, Yanhong Sun, Jie Xing

**Affiliations:** School of Pharmaceutical Science, Shandong University, 44 West Wenhua Road, Jinan, 250012 People’s Republic of China

**Keywords:** Naphthoquine, Malaria, Gender, Pharmacokinetics, Antiplasmodial activity

## Abstract

**Background:**

Naphthoquine (NQ) is a suitable partner anti-malarial for the artemisinin-based combination therapy (ACT), which is recommended to be taken orally as a single-dose regimen. The metabolism of NQ was mainly mediated by CYP2D6, which is well-known to show gender-specific differences in its expression. In spite of its clinical use, there is limited information on the pharmacokinetics of NQ, and no data are available for females. In this study, the effect of gender on the pharmacokinetics and antiplasmodial efficacy of NQ in rodents was evaluated. The underlying factors leading to the potential gender difference, i.e., plasma protein binding and metabolic clearance, were also evaluated.

**Methods:**

The pharmacokinetic profiles of NQ were investigated in healthy male or female rats after a single oral administration of NQ. The antiplasmodial efficacy of NQ was studied in male or female mice infected with *Plasmodium yoelii*. The recrudescence and survival time of infected mice were also recorded after drug treatment. Plasma protein binding of NQ was determined in pooled plasma collected from male or female mice, rat or human. In vitro metabolism experiments were performed in the liver microsomes of male or female mice, rat or human.

**Results:**

The results showed that the gender of rats did not affect NQ exposure (AUC_0–t_ and C_max_) significantly (*P *> 0.05). However, a significant (*P *< 0.05) longer t_1/2_ was found for NQ in male rats (192.1 ± 47.7), compared with female rats (143.9 ± 27.1). Slightly higher but not significant (*P *> 0.05) antiplasmodial activity was found for NQ in male mice (ED_90_, 1.10 mg/kg) infected with *P. yoelii*, compared with female mice (ED_90_, 1.67 mg/kg). The binding rates of NQ to plasma protein were similar in males and females. There was no metabolic difference for NQ in male and female mice, rat or human liver microsomes.

**Conclusions:**

These results indicated that the pharmacokinetic profiles of NQ were similar between male and female rats, except for a longer t_1/2_ in male rats. The difference was not associated with plasma protein binding or hepatic metabolic clearance. Equivalent antiplasmodial activity was found for NQ in male and female mice infected with *P. yoelii*. This study will be helpful for the rational design of clinical trials for NQ.

## Background

To avoid rapid drug resistance, it is recommended to only use artemisinins as part of a combination with other drugs, i.e., the artemisinin drug acts for a rapid clearance of most *Plasmodium falciparum* and the concomitant partner anti-malarial with a prolonged half-life is responsible for eliminating residual parasites [1, 2]. As parasite has developed resistance to artemisinin drugs in Southern Asia, the contribution of the partner drug gets more attention. Several clinically used partner anti-malarials are 4-aminoquinolines, such as piperaquine, amodiaquine and naphthoquine (NQ) [3–5]. They have been suggested to act on the blood stages of the parasite’s life cycle and inhibit haemozoin formation [6, 7]. As a new generation of artemisinin-based combinations, NQ is used in combination with artemisinin (ARCO^®^), which is recommended to be taken orally as a single-dose regimen to improve patient compliance and avoid rapid development of parasite resistance [8]. The combination of artemisinin-NQ shows a high cure rate (98.1%) and a short parasite clearance time (34.6 ± 14.3 h) in patients [9, 10]. NQ also displayed a remarkable antiplasmodial activity against *P. falciparum* (IC_50_ of 8.0 nM) ex vivo and *Plasmodium berghei* in mice (ED_90_ of 0.63 mg/kg) [11, 12].

Although artemisinin-based combination therapy (ACT) is safe and efficacious to treat uncomplicated malaria, tolerability and efficacy might vary between different people, including children, male adults, pregnant-women and non-pregnant women. It was shown that parasites always took longer to clear in female patients [13]. Gender-related differences in pharmacokinetics have frequently been considered as potentially important determinants for the clinical effectiveness of drug therapy [14]. Despite more than 10 years of clinical use, there is limited information on the pharmacokinetics of NQ and no data are available for females. NQ was absorbed completely with an oral bioavailability > 90% in healthy male adults [15]. The time to peak plasma concentration (t_max_) of NQ was around 2–4 h with an extremely long elimination half-life (t_1/2_, 250–300 h) [16]. The major metabolic pathways of NQ were hydroxylation and *N*-oxidation, which were mainly mediated by CYP2D6, an enzyme well-known for its polymorphism and gender-specific differences in expression [17]. NQ was widely distributed in the tissue (liver, kidney and lung) and predominantly excreted from urine [9].

In this study, the effect of gender on the pharmacokinetics and antiplasmodial potency of NQ was investigated. The underlying factors, i.e., plasma protein binding and metabolic clearance, leading to the potential gender difference were also evaluated in mouse, rat and human.

## Methods

### Chemicals and reagents

Naphthoquine phosphate was purchased from Kunming Pharmaceutical Corporation (KPC, purity > 98.0%, Yunnan, China). Chloroquine (CQ) was purchased from the National Institutes for Food and Drug Control (purity > 99.0%, Beijing, China). Amodiaquine was purchased from Sigma-Aldrich (St. Louis, MO, USA). Other chemicals used were purchased from Sigma-Aldrich or Fisher Scientific.

### Parasite strain

The murine malaria parasite *Plasmodium yoelii* was obtained from the Malaria Research and Reference Reagent Resource Center (MR4) as a part of the BEI Resources Repository, National Institute of Allergy and Infectious Diseases, National Institute of Health.

### Animal handling

ICR mice (20–25 g) and Wistar rats (200–220 g) were supplied by the Laboratory Animal Centre of Shandong University (Grade II, Certificate No. SYXK2013-0001). The experimental protocol was approved by the University Ethics Committee and conformed to the “Principles of Laboratory Animal Care” (NIH publication no. 85-23, revised 1985). Laboratory animals were fasted for 12 h before drug administration and for a further 2 h after dosing. Water was freely available during experiments.

### In vivo antiplasmodial activity

Male or female ICR mice were treated intraperitoneally (*i.p.)* with 1 × 10^7^ red blood cells (RBCs) infected with *P. yoelii*. The positive model drug (CQ; dissolved in 0.03% acetic acid) was orally administered at 24, 48 and 72 h post-infection. To simulate the clinical use of NQ as a single-dose regimen, the tested drug NQ (dissolved in 0.03% acetic acid) was orally given at 24 h post-infection. Efficacy was carried out using five different dose levels with nine mice at each level. Parasitaemia was assessed by microscopic examination of Giemsa-stained blood smears on day 4 post-infection. The 50% or 90% growth inhibitory doses (ED_50_ or ED_90_, respectively) of NQ were an average of three independent measurements (three mice in each dose group). Mice without the parasitaemia were considered fully cured.

### Pharmacokinetic study

Healthy male or female rats (n = 7 for each group) were given a single oral dose of NQ (40 mg/kg; dissolved in 0.03% acetic acid). Blood samples (150 μL) were withdrawn before dosing and at 0.5, 1, 2, 3, 4, 6, 8, 12, 24, 36, 48, 60, 72, 84, 96, 120, 144, 168, 240, 336, 504, 672 and 840 h after dosing. Heparinized blood samples were centrifuged and plasma samples were stored at − 20 °C until analysis.

### Quantification of NQ

Plasma samples were subjected to a protein precipitation extraction process. In brief, 20 μL of rat plasma was mixed with 4 μL of 0.03% hydrochloride acid, followed by addition of 10 μL of internal standard (IS, amodiaquine, 200 ng/mL) and 200 μL of acetonitrile. The samples were mixed and centrifuged at 14,000 rpm for 20 min. The supernatant was evaporated to dryness at 45 °C in a speedVac concentrator, and the residue was reconstituted in 150 μL of the initial mobile phase before LC–MS/MS analysis. For calibration preparation, 20 μL of drug-free plasma was mixed with 4 μL of stock solution (prepared in 0.03% hydrochloride acid), 10 μL of IS and 200 μL of acetonitrile. The mixture was treated as above. Matrix-matched calibration standards were obtained with concentrations of 1.0–400.0 ng/mL for NQ. The analytical method was fully validated according to the Guidelines on bioanalytical method validation drafted by US Food and Drug Administration (2013), which included selectivity, linearity, accuracy and precision, matrix-effect, recovery, dilution integrity, carryover and stability.

An LC–MS method was applied for quantification of NQ on an API5500 Q-Trap triple quadrupole mass spectrometer (AB SCIEX, Concord, Ontario, Canada) equipped with a TurboIonSpray source. The chromatographic separation was achieved on a Poroshell 120 SB-C_18_ column (100 × 4.6 mm i.d., 2.7 μm, Agilent Technologies) at 40 °C. The mobile phase consisted of (A) acetonitrile and (B) 0.2% formic acid and 0.05% trifluoroacetic acid, delivered at a flow rate of 0.6 mL/min. The HPLC gradient system started with 30% A for 0.5 min and linearly increased to 90% A in 2.5 min, followed by a decrease to 30% A prior to column re-equilibration. The electrospray ion source was operated in the positive ionization mode. The ionization voltage was + 3.5 kV and the source temperature was set at 550 °C. Nitrogen was used as the curtain gas (40 psi), nebulizer gas (GS1, 55 psi) and turbo gas (GS2, 55 psi). The multiple reaction monitoring (MRM) transitions were *m/z* 410.0 → 337.1 and *m/z* 356.0 → 283.1 for NQ and IS, respectively.

### Plasma protein binding

Plasma protein binding (PPB) of NQ (0.1 and 1.0 μg/mL) was determined in pooled plasma collected from female or male mice, rat or human, using the ultrafiltration method. Briefly, stock solution of NQ was diluted with blank plasma to achieve the test concentrations. Incubations were performed in a shaking water bath at 37 °C for 1 h to allow equilibration. Plasma samples were loaded into the Ultra centrifugal filters (Millipore, USA) with 10 kDa molecular weight cutoff, and the filtrate was centrifuged at 6000 rpm for 20 min at 37 °C. Phosphate buffered saline (PBS) was used to test non-specific binding (NSB). The NSB was calculated according to the equation:

$$ {\text{NSB}}\, = \,\left( {{\text{C}}_{\text{BD}} - {\text{C}}_{\text{BF}} } \right)/{\text{C}}_{\text{BD}} , $$

where C_BD_ was the total drug concentration in PBS before centrifugation and C_BF_ was the drug concentration in the PBS filtrate after centrifugation. The PPB was calculated based on the equation:

$$ {\text{PPB}}\% \, = \, 100\, \times \,\left( { 1 - {\text{C}}_{\text{SF}} /\left[ {\left( { 1 - {\text{NSB}}} \right)\, \times \,{\text{C}}_{\text{SD}} } \right]} \right), $$

where C_SF_ was the NQ concentration in the plasma ultrafiltrate and C_SD_ was the nominal plasma concentration. All drug concentrations were determined by LC–MS/MS.

### Metabolic clearance of NQ in liver microsomes

Pooled liver microsomes derived from male or female mice, rat or human were purchased from RILD Research Institutes for Liver Diseases (Shanghai, China). NQ (10 μM) was incubated with pooled male or female liver microsomes derived from three species, i.e., mice (MLM), rat (RLM) and human (HLM) (1 mg/mL) in potassium phosphate buffer (0.1 M, pH 7.4) and NADPH (1 mM) at 37 °C for 1 h. The incubation was initiated by adding NADPH and stopped by adding two volumes of cold acetonitrile. After centrifugation at 3000*g* for 10 min, the supernatant was dried under N_2_ at 45 °C and then reconstituted with initial mobile phase. An aliquot of the reconstituted sample was analyzed by LC–MS/MS. The in vitro intrinsic clearance (CL_int, in vitro_) was calculated according to the equation:

$$ {\text{CL}}_{{{\text{int}},{\text{in}}\;{\text{vitro}}}} \, = \,\left( {0. 6 9 3/{\text{t}}_{ 1/ 2} } \right)\, \times \,\left( { 1/{\text{C}}_{\text{protein}} } \right), $$

where C_protein_ was the protein concentration.

### Data analysis

Drug susceptibility was analysed by a nonlinear regression of logarithmically transformed concentrations. The doses that inhibited parasite growth by 50% (ED_50_) and 90% (ED_90_) were determined for NQ against *P. yoelii* in infected male or female mice. The peak plasma concentration (C_max_) and the time to peak concentration (t_max_) were obtained from experimental observations. The other pharmacokinetic parameters were analyzed by use of a non-compartmental model and the program TOPFIT (version 2.0; Thomae GmbH, Germany). The area under the plasma concentration–time curve (AUC_0–t_) was calculated using the linear trapezoidal rule to approximately the last point. Total oral body clearance (CL/F) was calculated as dose/AUC_0–t_. The terminal elimination half-life (t_1/2_) was estimated by log-linear regression in the terminal phase using an average of five observed concentrations.

Results were expressed as mean ± SD. Comparison of the pharmacokinetic parameters (AUC_0–t_ and C_max_) were performed after logarithmic transformation, and the mean changes in pharmacokinetic parameters among different groups were compared using Student’s *t*-test, which were performed with SPSS (version 19.0, SPSS Inc., Chicago, IL, USA). The comparison of t_max_ for the different treatment groups was performed using the Wilcoxon signed-rank test. The acceptable level of significance was established at *P *< 0.05. A greater than 1.5 increase in AUC_0–t_ or antiplasmodial activity (ED_50_ or ED_90_), relative to the control, was defined to be significant.

## Results

### Bioanalysis of NQ in plasma

No endogenous interfering peak derived from biological matrices was observed in the MRM channel of each analyte (Additional file 1: Fig. S1). The linear regression curves were obtained over the concentration ranges of 1.0–400.0 ng/mL for NQ. The intra- and inter-day accuracy and precision for QC samples were in acceptable ranges. The plasma matrix was negligible under the current conditions, and the results showed sufficient extraction efficiency (80–95%) for NQ and IS. Good stability of NQ was found during sample preparation and analysis, as well as under different storage conditions.

### The effect of gender on the pharmacokinetics of NQ in rats

**Fig. 1:**
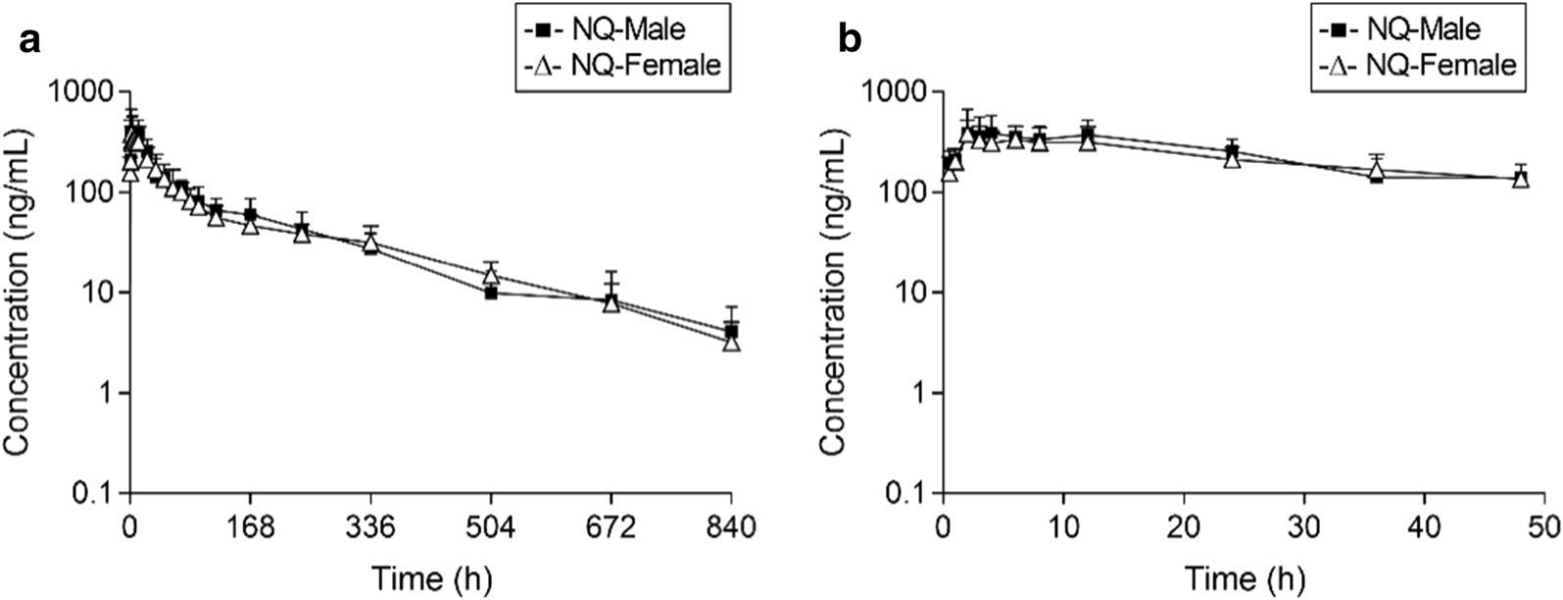
Mean (+ SD) plasma concentration–time profiles of naphthoquine (NQ) in male and female rats (n = 7 for each group) following a single oral administration of NQ (40 mg/kg). **a** From 0 to 840 h; **b** from 0 to 50 h

The mean plasma concentration–time profiles of NQ in male or female rats after a single oral dose of NQ (40 mg/kg) are displayed in Fig. 1. The pharmacokinetic parameters are shown in Table 1.

**Table 1:** The main pharmacokinetic parameters (mean ± SD) of naphthoquine (NQ) in male and female rats (n = 7 for each group) after a single oral administration of NQ (40 mg/kg)

Parameters	Male	Female
AUC_0–t_ (μg h/mL)	36.12 ± 11.25	32.82 ± 6.65
AUC_0–∞_ (μg h/mL)	37.44 ± 11.83	32.70 ± 7.06
C_max_ (ng/mL)	504.1 ± 206.6	438.1 ± 114.5
T_max_ (h)	5.4 (2.0–12.0)	3.1 (2.0–6.0)
t_1/2_ (h)	192.1 ± 41.2	143.9 ± 27.1*
MRT (h)	162.6 ± 47.7	173.1 ± 26.9
CL/F (L/h/kg)	1.22 ± 0.45	1.27 ± 0.27
Vz (L/kg)	316.7 ± 109.1	285.4 ± 104.8

* *P *< 0.05 (vs. male rats)

The pharmacokinetic property of NQ in male rats was characterized by multiple concentration peaks during the absorption phase and a multiphasic disposition with a long terminal half-life (192.1 ± 41.2 h). In addition, the variability in t_max_ was high (from 2.0 to 12.0 h). In comparison, the pharmacokinetic profile of NQ was similar in female rats, including the exposure (AUC_0–t_ and C_max_), t_max_ (around 3 h), CL/F and V_z_. However, a significantly shorter t_1/2_ (143.9 ± 27.1 h) was obtained for female rats.

### The effect of gender on the antiplasmodial activity of NQ in infected-mice

The in vivo antiplasmodial activity of NQ was assessed in male or female mice infected with *P. yoelii* after an oral administration of NQ. The antiplasmodial activity of NQ (ED_50_ and ED_90_) is shown in Table 2. The dose–response curves for NQ are shown in Additional file 2: Fig. S2, and the representative Giemsa-staining images of parasites in different drug-treated groups are shown in Additional file 3: Fig. S3.

**Table 2:** Antiplasmodial activity of naphthoquine (NQ) in male and female mice infected with *Plasmodium yoelii*

Treatment	ED_50_ (mg/kg)	ED_90_ (mg/kg)
CQ (male)	0.76 ± 0.16	2.18 ± 0.41
NQ (male)	0.71 ± 0.08	1.10 ± 0.11
NQ (female)	0.94 ± 0.12	1.67 ± 0.47

Chloroquine (CQ) was used as a positive model drug. In vivo experiments consisted of 5 doses, with 9 mice/dose. ED_50_ and ED_90_ were calculated by Prism (GraphPad) software from a best-fit curve

The infected mice treated with the positive-control drug CQ showed remarkable anti-malarial activity (ED_90_, 2.18 mg/kg), and a dose of 4.0 mg/kg/day CQ diminished *P. yoelii* parasitaemia by 98% on day 4. NQ dose–response testing showed an ED_50_ value of 0.71 mg/kg and an ED_90_ of 1.10 mg/kg in male mice. A non-significantly (*P *> 0.05) lower anti-malarial activity (ED_90_, 1.67 mg/kg) was found for NQ in female mice.

**Fig. 2:**
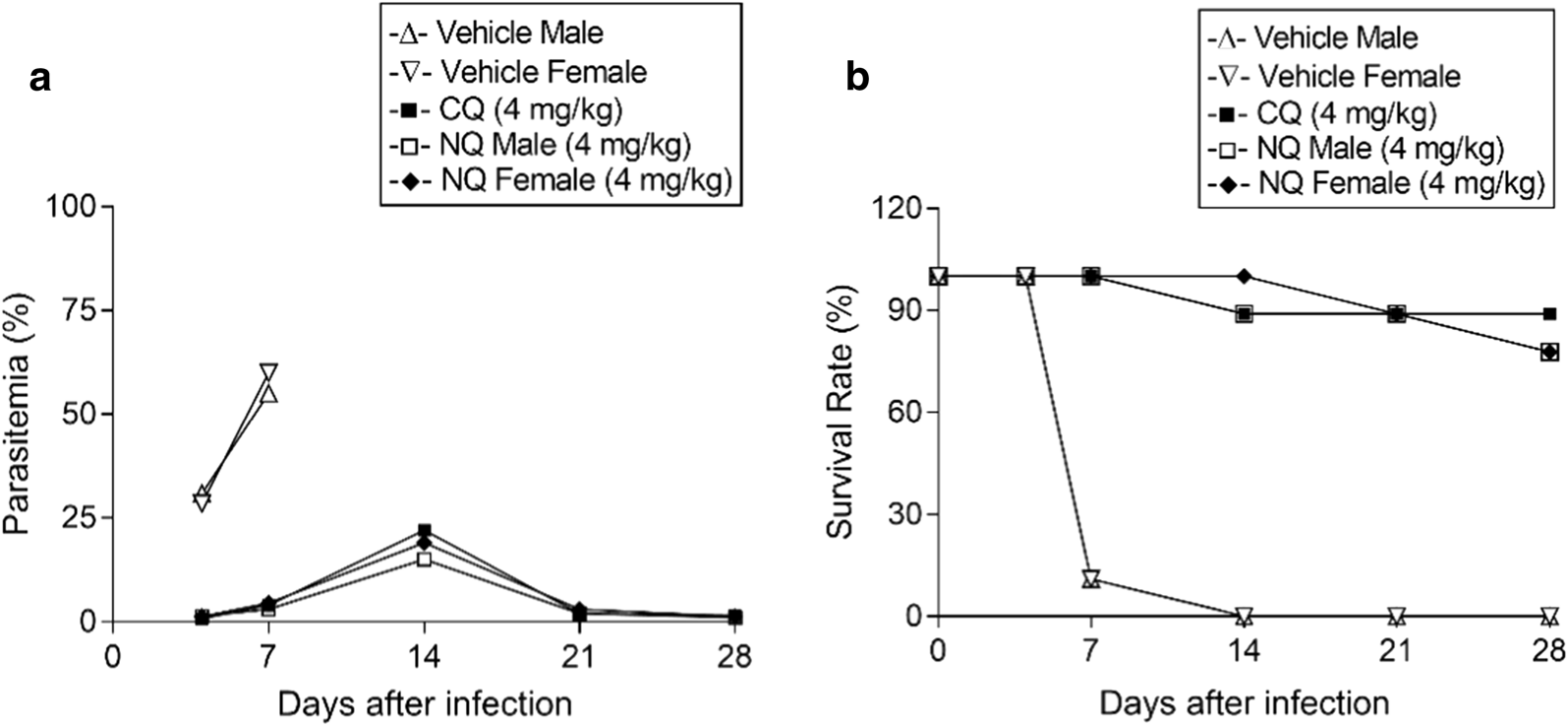
Mean parasitaemia (**a**) and survival rate (**b**) of *P. yoelii*-infected male or female mice (n = 9 for each dose) treated with vehicle control, chloroquine (CQ, positive model drug; 4 mg/kg), or naphthoquine (NQ, 4 mg/kg)

To test whether NQ could completely clear infection after a single oral dose, the recrudescence, survival time and mortality of infected-mice were also recorded (Fig. 2 and Additional file 4: Table S1). When CQ was given to infected mice (4.0 mg/kg), the survival rate of infected mice was 88.9% on day 28. A single dose treatment with NQ (4.0 mg/kg) reduced the *P. yoelii* parasitaemia to less than 1.0% on day 4 post-dosing, and 7 out of 9 mice could survive for at least 28 days without parasitaemia. Male and female mice showed comparable efficacy, in terms of survival rate and the parasitaemia at each dose group.

A single-dose toxicity evaluation was also performed for NQ at the dose as high as tenfold the ED_50_, and the general measures of animal well-being (weight and locomotor activity) did not show toxicity.

### The effect of gender on the PPB of NQ

The PPB of NQ was high (> 80%) with no species or gender difference. In addition, the PPB of NQ was not affected by drug concentrations tested (0.1 and 1.0 μg/mL). The PPB of NQ in male and female mice, rats, and humans are shown in Table 3.

**Table 3:** Plasma protein binding (%) of naphthoquine (NQ) in male and female mice, rats, and humans

Species	0.1 (μg/mL)	1.0 (μg/mL)
Mice (male)	82.55% ± 0.30%	82.55% ± 0.31%
Mice (female)	82.27% ± 0.30%	82.34% ± 0.41%
Rat (male)	82.57% ± 0.29%	82.44% ± 0.35%
Rat (female)	82.54% ± 0.24%	82.41% ± 0.30%
Human (male)	82.47% ± 0.32%	82.52% ± 0.31%
Human (female)	82.42% ± 0.22%	82.48% ± 0.24%

### The effect of gender on the metabolic clearance of NQ in liver microsomes

**Fig. 3:**
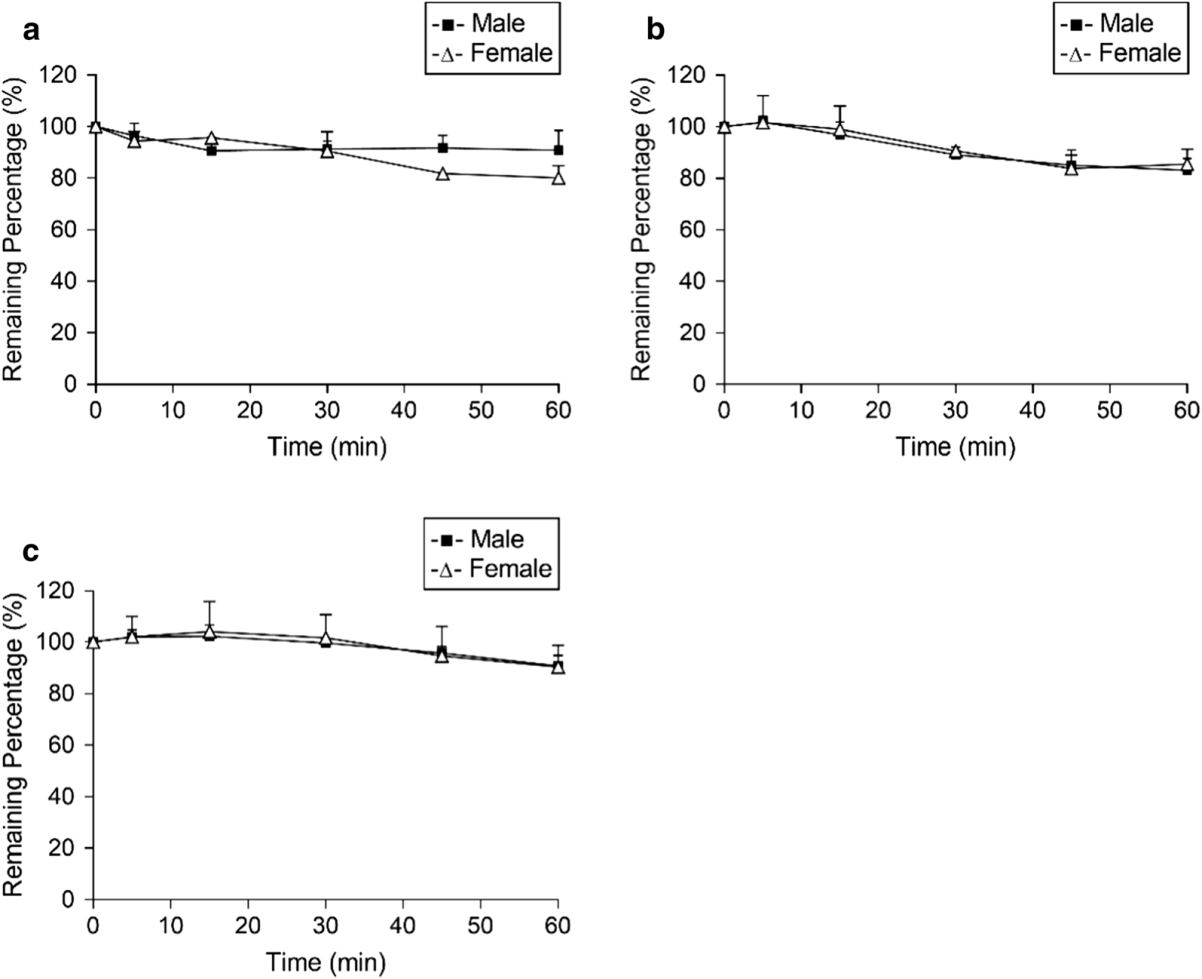
Metabolic depletion profiles of naphthoquine (NQ) in liver microsomes of male or female mice (**a**), rats (**b**), or humans (**c**)

The metabolic depletion profiles of NQ in liver microsomes are shown in Fig. 3, and the calculated t_1/2_ and CL_int in vitro_ are shown in Table 4. Incubation of NQ (10 μM) at 37 °C for 60 min resulted in 10% and 20% depletion of NQ in male and female MLM, respectively. The t_1/2_ of NQ in male MLM was 811.3 ± 211.7 min, and its corresponding CL_int in vitro_ was 0.90 ± 0.26 μL/min/mg. The metabolic clearance of NQ in female MLM did not show any difference (*P *> 0.05). Approximately 85% of NQ remained after incubation for 60 min in RLM. The t_1/2_ and the CL_int in vitro_ of NQ were similar in male and female RLM. For HLM, around 90% of NQ remained, and the metabolic depletion profiles were similar in male and female HLM, in terms of t_1/2_ and CL_int in vitro_.

**Table 4:** Metabolic clearance of naphthoquine (NQ) in the liver microsomes of male and female mice, rats, and humans

Species	t_1/2_ (min)	CL_int, in vitro_ (μL/min/mg)
Mice (male)	811.3 ± 211.7	0.90 ± 0.26
Mice (female)	436.7 ± 111.2	1.67 ± 0.47
Rat (male)	490.9 ± 180.3	1.53 ± 0.50
Rat (female)	490.0 ± 190.5	1.57 ± 0.60
Human (male)	930.4 ± 200.4	0.77 ± 0.15
Human (female)	1089.0 ± 514.4	0.80 ± 0.52

## Discussion

Despite the emergence of parasite resistance to artemisinin drugs in Southeast Asian, artemisinin drug-based combination therapy (ACT) is still the first-line treatment for malaria. The traditional ACT medicines include dihydroartemisinin plus piperaquine, artemether plus lumefantrine, artesunate plus mefloquine and artesunate plus amodiaquine, which are recommended by the World Health Organization (WHO) as a 3-day treatment. A single dose therapy with artemisinin plus NQ (ARCO) is also used in many tropical countries due to its comparable efficacy and cost-effectiveness [8–10, 15]. Recent work has shown that the effectiveness of these anti-malarials dependents on various factors, such as ethnicity, gender, previous infection, and type of treatment [18–20]. The dosing regimens of artemisinin drugs and several partner drugs in the four traditional ACT medicines have been optimized based on a comprehensive understanding of their pharmacokinetics and clinical responses, including among vulnerable groups, i.e., (non)-pregnant women and children under 5 years infected with uncomplicated malaria. However, the current dose regimen of NQ more relied on the clinical observations. Limited information was available for the pharmacokinetics of NQ except two studies performed in healthy male adults and infected children, which showed inconsistent results for the food effect [16, 21]. No pharmacokinetic data of NQ was available for females, even in lab animals. To achieve sustainable use of NQ, collective efforts should be concerned to understand the inter-individual differences in response toward NQ therapy. In a previous study, the metabolism of NQ was mainly mediated by CYP2D6, which is known for its polymorphism and a gender-specific difference in its expression [17]. Women have been found to have a higher CYP2D6 activity [22]. To reveal factors leading to the inter-individual differences in NQ pharmacokinetics and clinical response, the effect of gender on the pharmacokinetics and antiplasmodial efficacy was evaluated in the present study. In addition, the probable factors, i.e., plasma protein binding and metabolic clearance, leading to the potential gender difference were also evaluated.

In this study, the pharmacokinetic profiles of NQ (AUC_0–t_ and C_max_) were similar in female and male rats. Multiple concentration peaks were found for NQ, and the variability in t_max_ (2–12 h) was high, possibly reflecting the inter-individual variability in gastric emptying time. A difference in the terminal slope was observed for NQ between female and male rats. A significantly longer t_1/2_ was obtained for NQ in male rats (192.1 h vs. 143.9 h in female rats), which was indicative of an impact of gender on NQ disposition. This may arise from differences in plasma protein condition, organ blood flow, and expression levels of metabolizing enzymes and transporters [23, 24]. Since NQ was widely distributed in the tissue (liver, kidney and lung), gender differences in NQ distribution were expected in rats. Females generally have a lower bodyweight, with lower organ size and blood flow [25]. The effect of gender on the pharmacokinetic has been shown as anti-malarial drug-dependent. Increased clearance of dihydroartemisinin has been found in male patients, whereas female patients had higher oral clearance for artemisinin [19, 26]. Primaquine and (−)-mefloquine displayed a significantly higher exposure in healthy women [27–29].

Although gender disparity in pharmacokinetics has been identified for many drugs, gender differences in clinical responses may be only subtle. In this study, the antiplasmodial activity of NQ against *P. yoelii* in male mice (ED_90_, 1.10 mg/kg) was not-significantly (*P *> 0.05) higher than female mice (ED_90_, 1.67 mg/kg). Furthermore, male and female mice showed comparable efficacy, in terms of survival rate and the parasitaemia at each dose group.

Research with a gender perspective presents a high degree of complexity, and the inclusion of gender variability in clinical experiments brings many methodological questions. A gender-specific attention to pre-analytical evaluation could promote the translation from the bench to bedside, with an adequate gender-specific clinical development plan. Furthermore, gender-specific pre-clinical pharmacological testing will enable comprehensive assessment of pharmacokinetic and pharmacodynamic actions of drugs. In a previous study, species similarity has been observed in NQ metabolism between human and rodents [17]. The major metabolic pathways included hydroxylation and *N*-oxidation, which were mainly mediated by CYP2D6. The rat and human CYP2D isoforms share a high sequence identity (> 70%) and similar substrate specificities [30, 31]. In the present study, rat was selected as the laboratory animal in the pharmacokinetic study for the convenience of continuous blood sampling. Due to multiple concentration peaks of NQ and inconsistent blood sampling, mice were excluded from the pharmacokinetic evaluation. To avoid the effect of recovery from malaria infection on hepatic clearance of NQ, healthy rats were used in the pharmacokinetic study. To evaluate the antiplasmodial efficacy, mice infected with *P. yoelii* were selected as a standard animal model for the pre-clinical evaluation of anti-malarials.

Except for a longer t_1/2_ in male rats, no effects of gender on the pharmacokinetics and antiplasmodial activity of NQ were found in rodents. It will be valuable to investigate whether the obtained results were also predictive of the behaviour of NQ in human. In general, small animals tend to eliminate drugs more rapidly than human beings when compared on a weight-normalized basis. Compared with the pharmacokinetic data obtained in the present study, a longer t_1/2_ has been found for NQ in healthy adults (250–300 h) [16]. The factors that may lead to inaccurate determination of t_1/2_ were excluded, which includes the analytical sensitivity and short sampling time. Moreover, it is important to realize that humans differ from animals with regard to CYP isoform composition, expression and catalytic activities [30].

The mechanistic processes underlying gender-specific pharmacokinetics can be divided into physiological and molecular factors. Although gender does not affect albumin, the major drug-binding protein in plasma, α-acid glycoprotein is expressed slightly lower in females as a result of its known decrease in endogenous estrogen [32, 33]. Differences in lipoprotein concentrations might be present as well. In the present study, the binding rate of NQ to plasma protein was similar in males and females, which indicated that the PPB was gender independent. Major molecular factors involved in drug disposition include drug transporters and drug-metabolizing enzymes. Women tend to have higher CYP2D6 activity than men; however, the rat orthologue CYP2D1 has also been suggested as male dominant [22, 34]. In this study, the metabolic clearance of NQ was investigated in liver microsomes derived from mice, rats and humans. The substrate depletion method was used instead of metabolite formation, due to multiple metabolites of NQ formed via CYP2D6 with a minor contribution of several other CYP enzymes, i.e., CYP2C19 and CYP2C8. The results indicated that there was no metabolic difference for NQ in male and female mice, rat or human.

## Conclusions

These results indicated that the pharmacokinetic profiles of NQ were similar in male and female rats, except for a longer t_1/2_ in male rats. Equivalent antiplasmodial activity was found for NQ in male and female mice infected with *P. yoelii*. Furthermore, there were no gender differences in the degree of protein binding and hepatic metabolic clearance. This study will be helpful for the rational design of clinical trials for NQ.

## Supplementary information

**Additional file 1: Fig. S1.** Representative multiple reaction monitoring (MRM) chromatograms of: (A) a blank rat plasma sample, (B) a blank rat plasma sample spiked with NQ (2 ng/mL) and IS, (C) a rat plasma sample at 5.0 h after a single oral administration of NQ (40 mg/kg).

**Additional file 2: Fig. S2.** Representative dose response curves for chloroquine (CQ, positive model drug, A), naphthoquine (NQ) in male mice (B), and NQ in female mice (C). The parasite growth is measured in fluorescence units and normalized to the control values to give percentage growth.

**Additional file 3: Fig. S3.** Representative Giemsa-staining images of different groups from *P. yoelii* infected mice at day-4, including vehicle control in male (A), vehicle control in female (B), chloroquine (CQ, positive control, 1 mg/kg, C), naphthoquine in male (1 mg/kg, D), and naphthoquine in female mice (1 mg/kg, E). Red arrows indicated the *P. yoelii*-infected red blood cells.

**Additional file 4: Table S1.** The survival rate of *P. yoelii*-infected male or female mice treated with naphthoquine (NQ).

## Data Availability

All data generated or analysed during this study are included in this published article and additional files.

## Abbreviations

ACT: Artemisinin-based combination therapy
NQ: Naphthoquine
CQ: Chloroquine
MLM: Mice liver microsome
RLM: Rat liver microsome
HLM: Human liver microsome
IS: Internal standard
MRM: Multiple reaction monitoring
PPB: Plasma protein binding
