# Supplementary material for: The gender-related variability in the pharmacokinetics and antiplasmodial activity of naphthoquine in rodents
Source: Malar J. 2020 Feb 13;19:71. doi: 10.1186/s12936-020-3153-8 (PMC7020547; doi:10.1186/s12936-020-3153-8)
Supplement: Supplementary file 1 — Additional file 1: Fig. S1. Representative multiple reaction monitoring (MRM) chromatograms of: (A) a blank rat plasma sample, (B) a blank rat plasma sample spiked with NQ (2 ng/mL) and IS, (C) a rat plasma sample at 5.0 h after a single oral administration of NQ (40 mg/kg). [file 12936_2020_3153_MOESM1_ESM.docx]

**(B)**

**(A)**

**(C)**


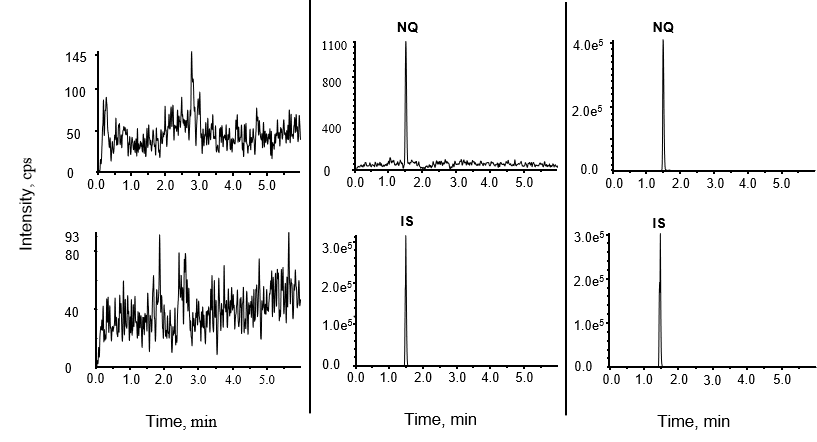


**Additional file 1: Fig. S1** Representative multiple reaction monitoring (MRM) chromatograms of: (A) a blank rat plasma sample, (B) a blank rat plasma sample spiked with NQ (2 ng/mL) and IS, (C) a rat plasma sample at 5.0 h after a single oral administration of NQ (40 mg/kg).
