# Supplementary material for: The gender-related variability in the pharmacokinetics and antiplasmodial activity of naphthoquine in rodents
Source: Malar J. 2020 Feb 13;19:71. doi: 10.1186/s12936-020-3153-8 (PMC7020547; doi:10.1186/s12936-020-3153-8)
Supplement: Supplementary file 2 — Additional file 2: Fig. S2. Representative dose response curves for chloroquine (CQ, positive model drug, A), naphthoquine (NQ) in male mice (B), and NQ in female mice (C). The parasite growth is measured in fluorescence units and normalized to the control values to give percentage growth. [file 12936_2020_3153_MOESM2_ESM.docx]

**(A) (B)**


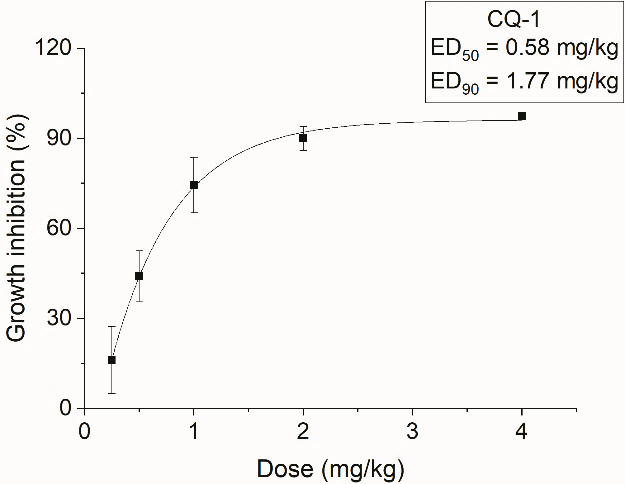

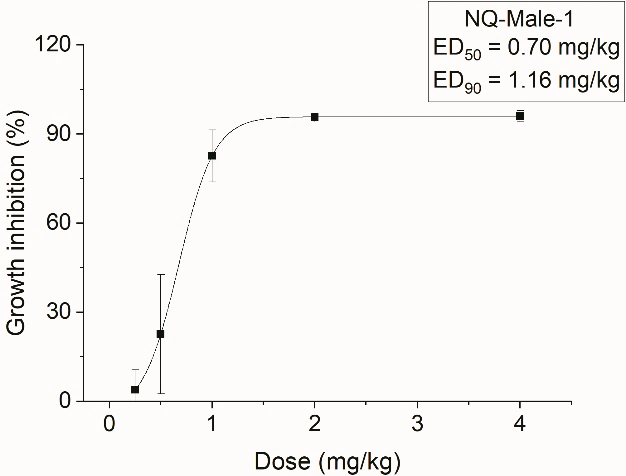


**(C)**


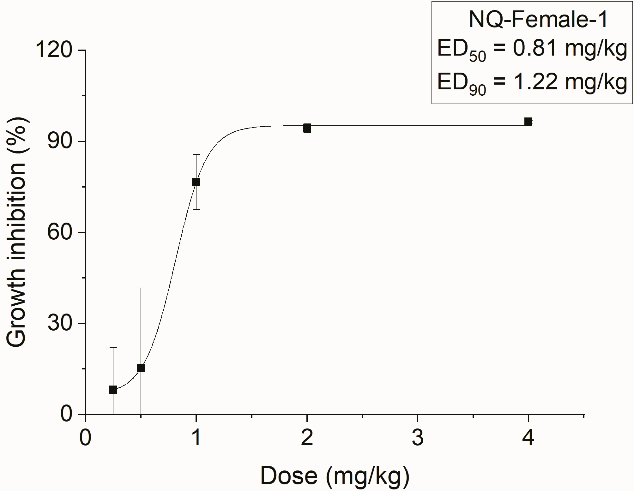


**Additional file 2: Fig. S2** Representative dose response curves for chloroquine (CQ, positive model drug, A), naphthoquine (NQ) in male mice (B), and NQ in female mice (C). The parasite growth is measured in fluorescence units and normalized to the control values to give percentage growth.
