# Supplementary material for: The gender-related variability in the pharmacokinetics and antiplasmodial activity of naphthoquine in rodents
Source: Malar J. 2020 Feb 13;19:71. doi: 10.1186/s12936-020-3153-8 (PMC7020547; doi:10.1186/s12936-020-3153-8)
Supplement: Supplementary file 3 — Additional file 3: Fig. S3. Representative Giemsa-staining images of different groups from P. yoelii infected mice at day-4, including vehicle control in male (A), vehicle control in female (B), chloroquine (CQ, positive control, 1 mg/kg, C), naphthoquine in male (1 mg/kg, D), and naphthoquine in female mice (1 mg/kg, E). Red arrows indicated the P. yoelii-infected red blood cells. [file 12936_2020_3153_MOESM3_ESM.docx]

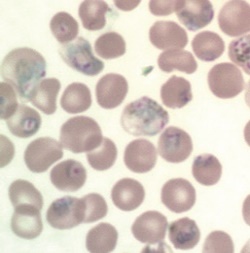

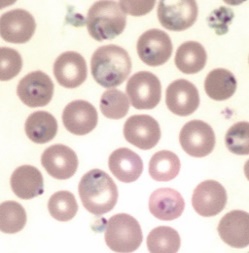

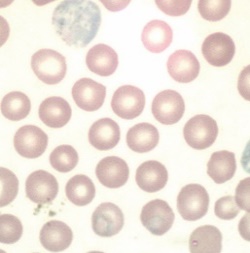

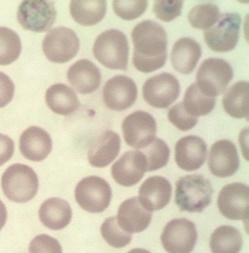

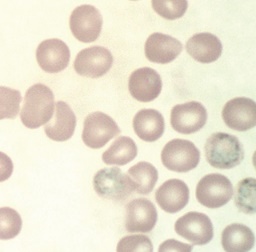


**(A)**

**(B)**

**(C)**

**(D)**

**(E)**

**Additional file 3: Fig. S3** Representative Giemsa-staining images of different groups from *P. yoelii* infected mice at day-4, including vehicle control in male (A), vehicle control in female (B), chloroquine (CQ, positive control, 1 mg/kg, C), naphthoquine in male (1 mg/kg, D), and naphthoquine in female mice (1 mg/kg, E). Red arrows indicated the *P. yoelii*-infected red blood cells.
