# Supplementary material for: The gender-related variability in the pharmacokinetics and antiplasmodial activity of naphthoquine in rodents
Source: Malar J. 2020 Feb 13;19:71. doi: 10.1186/s12936-020-3153-8 (PMC7020547; doi:10.1186/s12936-020-3153-8)
Supplement: Supplementary file 4 — Additional file 4: Table S1. The survival rate of P. yoelii-infected male or female mice treated with naphthoquine (NQ). [file 12936_2020_3153_MOESM4_ESM.docx]

**Additional file 4: Table S1** The survival rate of *P. yoelii*-infected male or female mice treated with naphthoquine (NQ).

|  | Dose (mg/kg) | Day-0 | Day-4 | Day-7 | Day-14 | Day-21 | Day-28 |
| --- | --- | --- | --- | --- | --- | --- | --- |
| Vehicle (male) |  | 100.0% | 100.0% | 11.1% | 0.0% | 0.0% | 0.0% |
| Vehicle (female) |  | 100.0% | 100.0% | 11.1% | 0.0% | 0.0% | 0.0% |
| CQ (male) | 0.25 (n=9) | 100.0% | 100.0% | 11.1% | 0.0% | 0.0% | 0.0% |
|  | 0.5 (n=9) | 100.0% | 100.0% | 66.7% | 0.0% | 0.0% | 0.0% |
|  | 1.0 (n=9) | 100.0% | 100.0% | 66.7% | 0.0% | 0.0% | 0.0% |
|  | 2.0 (n=9) | 100.0% | 100.0% | 100.0% | 77.8% | 33.3% | 33.3% |
|  | 4.0 (n=9) | 100.0% | 100.0% | 100.0% | 88.9% | 88.9% | 88.9% |
| NQ (male) | 0.25 (n=9) | 100.0% | 100.0% | 11.1% | 0.0% | 0.0% | 0.0% |
|  | 0.5 (n=9) | 100.0% | 100.0% | 22.2% | 0.0% | 0.0% | 0.0% |
|  | 1.0 (n=9) | 100.0% | 100.0% | 88.9% | 0.0% | 0.0% | 0.0% |
|  | 2.0 (n=9) | 100.0% | 100.0% | 100.0% | 100.0% | 33.3% | 33.3% |
|  | 4.0 (n=9) | 100.0% | 100.0% | 100.0% | 88.9% | 88.9% | 77.8% |
| NQ (female) | 0.25 (n=9) | 100.0% | 100.0% | 11.1% | 0.0% | 0.0% | 0.0% |
|  | 0.5 (n=9) | 100.0% | 100.0% | 0.0% | 0.0% | 0.0% | 0.0% |
|  | 1.0 (n=9) | 100.0% | 100.0% | 55.6% | 0.0% | 0.0% | 0.0% |
|  | 2.0 (n=9) | 100.0% | 100.0% | 100.0% | 66.7% | 44.4% | 44.4% |
|  | 4.0 (n=9) | 100.0% | 100.0% | 100.0% | 100.0% | 88.9% | 77.8% |

Chloroquine (CQ) was used as a positive model drug.
